# Neovestitol, an isoflavonoid isolated from Brazilian red propolis, reduces acute and chronic inflammation: involvement of nitric oxide and IL-6

**DOI:** 10.1038/srep36401

**Published:** 2016-11-07

**Authors:** Marcelo Franchin, David F. Colón, Marcos G. da Cunha, Fernanda V. S. Castanheira, André L. L. Saraiva, Bruno Bueno-Silva, Severino M. Alencar, Thiago M. Cunha, Pedro L. Rosalen

**Affiliations:** 1 Department of Physiological Sciences, Piracicaba Dental School, University of Campinas, Piracicaba, 13414 903, Brazil; 2 Department of Immunology, Ribeirão Preto Medical School, University of São Paulo, Ribeirão Preto, 14049-900, Brazil; 3 Department of Pharmacology, Ribeirão Preto Medical School, University of São Paulo, Ribeirão Preto, 14049-900, Brazil; 4 Dental Research Division, Guarulhos University, Guarulhos, São Paulo, CEP: 07023-070, Brazil; 5 Department of Agri-Food industry, Food and Nutrition, “Luiz de Queiroz” College of Agriculture, University of São Paulo, Piracicaba, 13418-900, Brazil

## Abstract

Isoflavonoids have been largely studied due to their distinct biological activities identified thus far. Herein, we evaluated the activity of neovestitol, an isoflavonoid isolated from Brazilian red propolis, in acute and chronic inflammation. As for acute inflammation, we found that neovestitol reduced neutrophil migration, leukocyte rolling and adhesion, as well as expression of ICAM-1 in the mesenteric microcirculation during lipopolysaccharide-induced acute peritonitis. No changes were observed in the levels of TNF-α, CXCL1/KC and CXCL2/MIP-2 upon pretreatment with neovestitol. The administration of an inducible nitric oxide synthase (iNOS) inhibitor abolished the inhibitory effects of neovestitol in neutrophil migration and ICAM-1 expression. Nitrite levels increased upon treatment with neovestitol. No effects of neovestitol were observed on the chemotaxis of neutrophils *in vitro*. As for chronic inflammation, neovestitol also reduced the clinical score and joint damage in a collagen-induced arthritis model. There was no change in the frequency of IL-17-producing TCD4+ cells. In addition, pretreatment with neovestitol reduced the levels of IL-6. These results demonstrate a potential anti-inflammatory activity of neovestitol, which may be useful for therapeutic purposes and/or as a nutraceutical.

The inflammatory process comprises a set of events involving the participation of cytokines (TNF-α), chemokines (CXCL1/KC and CXCL2/MIP-2), lipid mediators (leukotriene B4), cell adhesion molecules (ICAM-1), nitric oxide and immune cells. This process leads to vasodilation, increased vascular permeability and leukocyte recruitment^1^.

The occurrence of acute inflammation is related to a host defensive response to infectious agents and is characterized by the presence of neutrophils. On the other hand, chronic inflammation is a persistent phenomenon in which the presence of macrophages and lymphocytes becomes more evident^2^.

Although it is a host protective response, exacerbation of inflammation in some cases is directly related to tissue damage, thus requiring the use of anti-inflammatory drugs^3^. Furthermore, some inflammatory diseases such as rheumatoid arthritis frequently result from a chronic inflammatory process whose treatment with drugs is limited^4^.

Propolis has been used in folk medicine for decades for the treatment of various diseases^5^. In addition, it is also present in cosmetic formulations, toothpastes and food preservatives^6^,^7^,^8^. Brazilian propolis from *Apis mellifera* L. (Apidae) were classified into 13 types according to their chemical characteristics and the region surrounding the hives. Among the varieties, propolis “type 13” (also named as red propolis) has a typical reddish aspect^9^,^10^. Originally from Maceió, northeastern Brazil, this propolis has a peculiar presence of isoflavonoids in its composition. Among the identified isoflavonoids, neovestitol has shown antimicrobial, anticaries and antioxidant activity in preliminary studies^11^,^12^. The anti-inflammatory potential of neovestitol has also been reported^13^, however its activity in a chronic inflammation model, as well as the involvement of inflammatory mediators and adhesion molecules in its effect remain unexplored.

Thus, we evaluated the activity of neovestitol in the modulation of neutrophil migration, and also in the regulation of cytokines, nitric oxide and ICAM-1 adhesion molecule expression. Furthermore, we assessed the activity of neovestitol in an arthritis model (chronic inflammation), as well as the role of this compound in the regulation of inflammatory cytokines.

## Results

### Effects of neovestitol on acute inflammation

#### Neovestitol reduces migration, neutrophil adhesion and rolling and ICAM-1 expression in the peritoneal cavity of mice

**Figure 1:**
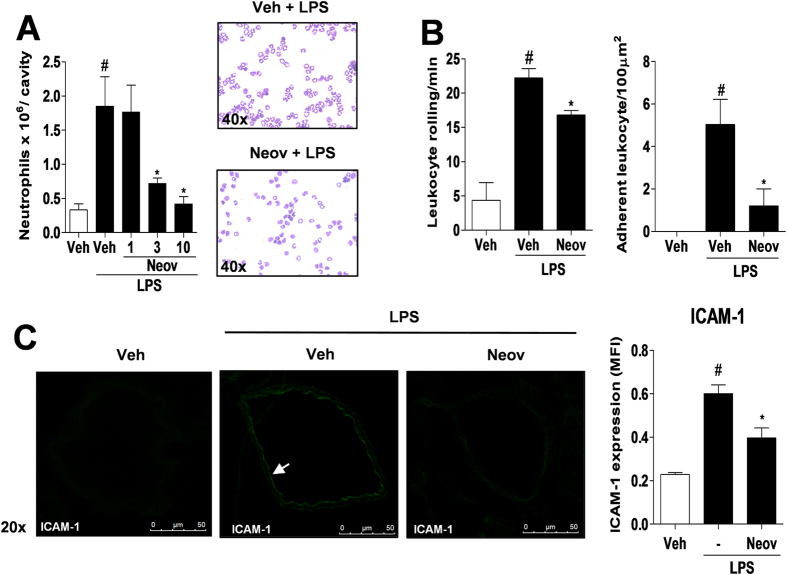
Neovestitol reduces LPS-induced peritoneal inflammation. C57BL/6 mice were pretreated with neovestitol (Neov) at the doses of 1, 3 or 10 mg/kg subcutaneously (sc) or vehicle (Veh) alone (1% DMSO in PBS), 30 min before intraperitoneal (ip) administration of LPS (300 ng/cavity). (**A**) Migration of neutrophils into the peritoneal cavity induced by LPS (300 ng/cavity) for 6 h. (**B**) Rolling and adhesion of leukocytes in the mesenteric microcirculation of mice pretreated with neovestitol (Neov) at the dose 10 mg/kg sc and stimulated with LPS (300 ng/cavity) for 2 or 4 h, respectively. (**C)** Expression of ICAM-1 in the mesenteric microcirculation of mice pretreated with neovestitol (Neov) at the dose 10 mg/kg sc and stimulated with LPS (300 ng/cavity) for 4 h. The data were expressed as mean ± SEM, with n = 4–5 per group. Symbols indicate statistical difference (P < 0.05, one-way ANOVA followed by Tukey’s test). ^#^P < 0.05 compared to vehicle group. *P < 0.05 compared to Veh + LPS group.

In order to assess the anti-inflammatory effects of neovestitol on acute inflammation we used an LPS-induced peritonitis model. In our study, we found that intraperitoneal (ip) injection of LPS induced significant neutrophil migration into the peritoneal cavity of mice as compared with the group that did not receive the LPS challenge (Veh) (Fig. 1A). Notably, subcutaneous treatment (sc) with neovestitol at 3 and 10 mg/kg reduced LPS-induced neutrophil migration (Fig. 1A). Next, we investigated the activity of neovestitol on leukocyte rolling and adhesion and on ICAM-1 expression in mesenteric microcirculation of LPS-challenged mice. According to the results, neovestitol at 10 mg/kg reduced leukocyte rolling and adhesion (Fig. 1B) and expression of ICAM-1 (Fig. 1C).

#### Neovestitol does not alter cytokine and chemokine levels in the peritoneal cavity of mice

**Figure 2:**
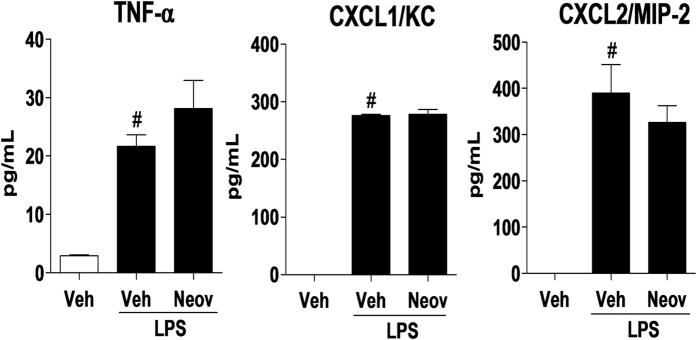
Neovestitol did not reduce the release of cytokines and chemokines in LPS-induced peritoneal inflammation. C57BL/6 mice were pretreated with neovestitol (Neov) at the dose 10 mg/kg subcutaneously (sc) or vehicle (Veh) alone (1% DMSO in PBS), 30 min before intraperitoneal (ip) administration of LPS (300 ng/cavity). Levels of TNF-α (1.5 h after stimulation with LPS), CXCL1/KC and CXCL2/MIP-2 (3 h after stimulation with LPS) in the peritoneal cavity of mice stimulated with LPS (300 ng/cavity). The data were expressed as mean ± SEM, with n = 4–5 per group. Symbols indicate statistical difference (P < 0.05, one-way ANOVA followed by Tukey’s test). ^#^P < 0.05 compared to Veh group. *P < 0.05 compared to Veh + LPS group.

Cytokines and chemokines play a key role in neutrophil migration in the inflammatory process, including the signaling for increasing adhesion molecules expression on the venular endothelium^14^. Thus, the activity of neovestitol was evaluated concerning TNF-α, CXCL1/KC and CXCL2/MIP-2 release in the peritoneal lavage. As a result, we found that pretreatment with neovestitol did not affect TNF-α, CXCL1/KC and CXCL2/MIP-2 levels in the peritoneal cavity of LPS-challenged mice (Fig. 2), therefore suggesting that the anti-inflammatory activity of neovestitol may be related to other pathways.

#### Neovestitol reduces neutrophil migration by a nitric oxide-dependent mechanism in the peritoneal cavity of mice

**Figure 3:**
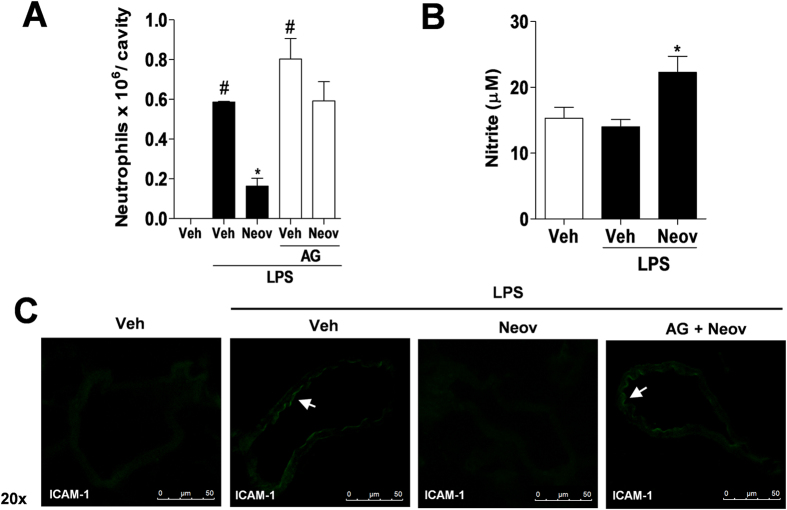
Neovestitol reduces LPS-induced peritoneal inflammation through a nitric oxide-dependent mechanism. C57BL/6 mice were pretreated with neovestitol (Neov) at the dose 10 mg/kg subcutaneously (sc) or vehicle (Veh) alone (1% DMSO in PBS), 30 min before intraperitoneal (ip) administration of LPS (300 ng/cavity). (**A**) Migration of neutrophils into the peritoneal cavity induced by LPS (300 ng/cavity) for 6 h in mice pretreated with aminoguanidine (AG) at 50 mg/kg sc. (**B**) Levels of nitrite in the peritoneal cavity of mice stimulated with LPS (300 ng/cavity) for 4 h. (**C**) Expression of ICAM-1 in the mesenteric microcirculation in mice stimulated with LPS (300 ng/cavity) for 4 h pretreated with AG (50 mg/kg) sc. The data were expressed as mean ± SEM, with n = 4–5 per group. Symbols indicate statistical difference (P < 0.05, one-way ANOVA followed by Tukey’s test). ^#^P < 0.05 compared to Veh group. *P < 0.05 compared to Veh + LPS group.

Nitric oxide plays a crucial role in modulating neutrophil migration in LPS-induced peritonitis in mice^15^,^16^. This study investigated the activity of neovestitol in neutrophil migration and expression of ICAM-1 against a pretreatment with an inducible nitric oxide synthase (iNOS) inhibitor (aminoguanidine 50 mg/kg). The results show that administration of aminoguanidine abolished the inhibitory effects of neovestitol on neutrophil migration (Fig. 3A) and ICAM-1 expression (Fig. 3C). It was also observed that pretreatment with neovestitol increased nitric oxide levels in the peritoneal cavity of mice (Fig. 3B). *In vitro* neovestitol at 30 μM did not reduce the expression of ICAM-1 (see Supplementary Fig. S1A) nor changed the viability of bEnd.3 cells (see Supplementary Fig. S1B). These findings support the hypothesis that part of the anti-inflammatory effect of neovestitol on peritonitis depends on the nitric oxide pathway.

#### Neovestitol does not affect chemotaxis of CXCL2/MIP-2-induced neutrophils

**Figure 4:**
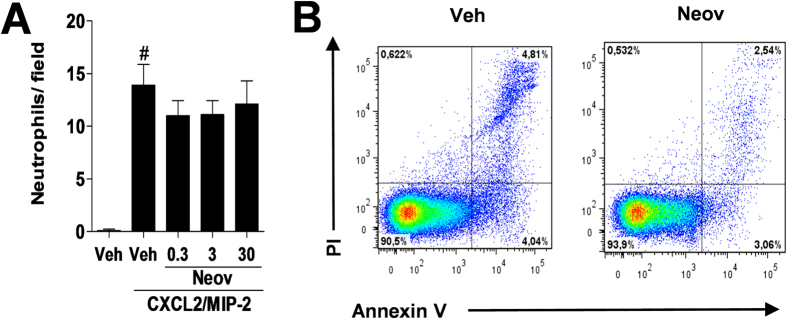
Neovestitol does not affect the chemotaxis of neutrophils *in vitro*. Neutrophils were pretreated with neovestitol (Neov) at the concentrations of 0.3, 3 or 30 μM or or vehicle (Veh) alone (1% DMSO in PBS), 30 min prior to stimulation with CXCL2/MIP-2 at 30 ng/mL. (**A**) Chemotaxis of neutrophils induced by CXCL2/MIP-2 (30 ng/mL) was evaluated after 1 h. (**B**) Neutrophil viability (annexin V and PI) after incubation with Neov at 30 μM for 1.5 h. The data were expressed as mean ± SEM, with n = 3–4 per group. Symbols indicate statistical difference (P < 0.05, one-way ANOVA followed by Tukey’s test). ^#^P < 0.05 compared to Veh group.

The activity of neovestitol on *in vitro* neutrophil chemotaxis was also evaluated. Chemotaxis occurs with the aid of various chemotactic factors, including the chemokine CXCL2/MIP-2 (IL-8)^17^. In our study, pretreatment with neovestitol at the concentrations of 0.3, 3 or 30 μM did not affect neutrophil chemotaxis induced by CXCL2/MIP-2 (Fig. 4A). In addition, there was no change in neutrophil viability during incubation with neovestitol at 30 μM (Fig. 4B).

### Effects of Neovestitol on chronic inflammation

#### Neovestitol reduces the clinical score and joint damage in mice with collagen-induced arthritis

**Figure 5:**
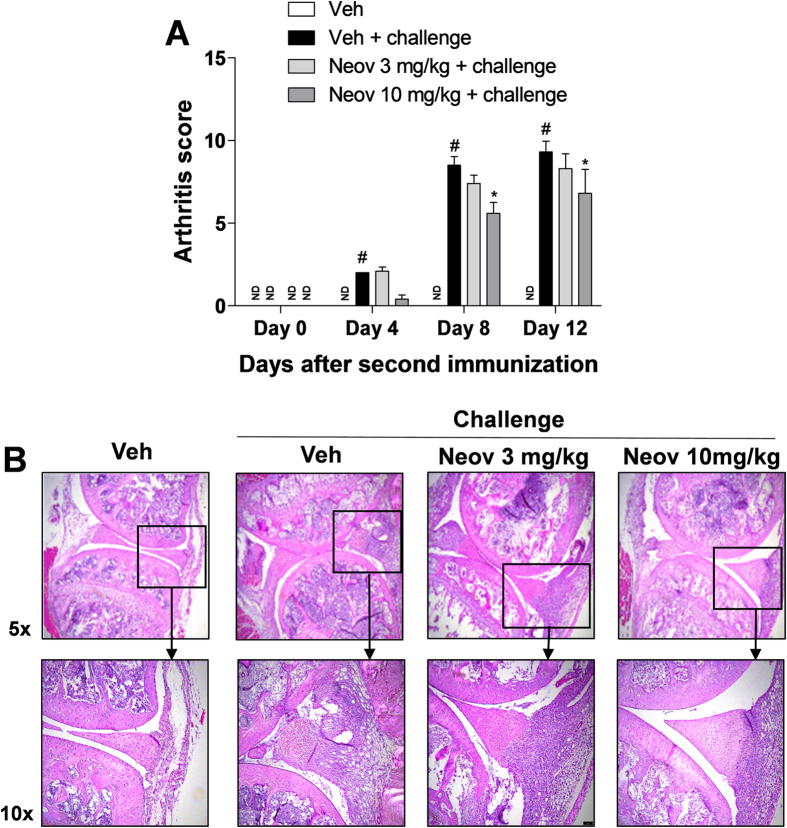
Neovestitol reduces joint inflammation in mice with arthritis. DBA1/J mice with arthritis were treated with neovestitol (Neov) at the doses of 3 or 10 mg/kg subcutaneously (sc) or vehicle (Veh) alone (1% DMSO in PBS) for 12 days after the boost. (**A**) Clinical score on days 0, 4, 8 and 12 after the second injection in the tail with type II collagen and CFA. (**B**) Histological analysis (5x and 10x magnification, optical microscope) of the joint using hematoxylin/eosin staining 12 days after the second challenge with type II collagen and CFA. The data were expressed as mean ± SEM, with n = 5–6 per group. Symbols indicate statistical difference (P < 0.05, two-way ANOVA followed by Bonferroni test). ^#^P < 0.05 compared to Veh group. *P < 0.05 compared to the Veh + Challenge group. The group of mice showing no increase in clinical scores is presented as “not detected” (ND).

The activity of neovestitol on collagen-induced arthritis in DBA-1/J mice (chronic inflammation) was investigated. After the second immunization with type II collagen and Complete Freud’s Adjuvant (CFA), there was a significant increase in the clinical scores of the animals on days 4, 8 and 12, as compared to the group that did not receive the challenge (Veh) (Fig. 5A). Administration of neovestitol at 10 mg/kg reduced the clinical scores of the animals with arthritis on days 8 and 12 after the second immunization (Fig. 5A). Nevertheless, such reduction was not observed at the dose of 3 mg/kg (Fig. 5A). Regarding the histological analysis of the joint after the last day of treatment (day 12), there was a decrease of joint damage (Fig. 5B) in the animals treated with neovestitol at the dose of 10 mg/kg.

#### Neovestitol does not affect the frequency of IL-17-producing TCD4+ cells in draining lymph nodes of mice with collagen-induced arthritis

**Figure 6:**
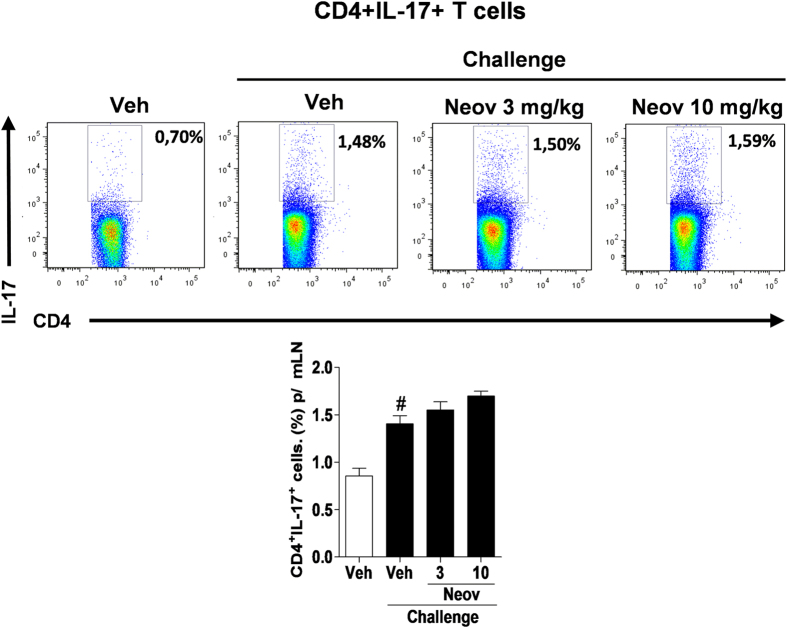
Neovestitol does not change the frequency of IL-17-producing TCD4+ cells in mice with arthritis. DBA1/J mice with arthritis were treated with neovestitol (Neov) at the doses of 3 or 10 mg/kg subcutaneously (sc) or vehicle (Veh) alone (1% DMSO in PBS) for 12 days after the boost. The frequency of IL-17-producing TCD4+ cells from draining lymph nodes was calculated 12 days after the second challenge with type II collagen and CFA. The data were expressed as mean ± SEM, with n = 5–6 per group. Symbols indicate statistical difference (P < 0.05, one-way ANOVA followed by Tukey’s test). ^#^P < 0.05 compared to Veh group.

Th17 lymphocytes are associated with chronic inflammation and development of various autoimmune diseases, including rheumatoid arthritis^18^. In the present study, we investigated the role of Th17 lymphocytes on the modulatory activity of neovestitol in arthritic mice. On day 12 after the second immunization (last day of treatment), the mice were killed and their draining lymph nodes were collected for analysis of the frequency of IL-17-producing TCD4+ cells. We found that administration of neovestitol at doses of 3 and 10 mg/kg in a 12-day period did not alter the frequency of IL-17-producing TCD4+ cells (Fig. 6).

#### Neovestitol reduces the release of IL-6 into the joint of mice with collagen-induced arthritis

**Figure 7:**
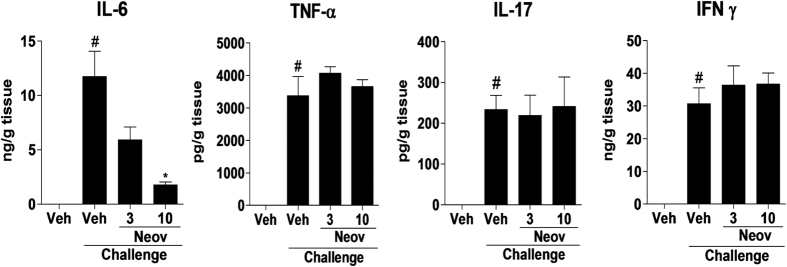
Neovestitol reduces the release of IL-6 in mice with arthritis. DBA1/J mice with arthritis were treated with neovestitol (Neov) at the doses of 3 or 10 mg/kg subcutaneously (sc) or vehicle (Veh) alone (1% DMSO in PBS) for 12 days after the boost. The levels of IL-6, TNF-α, IL-17 and IFN-γ in the joint 12 days after the second challenge with type II collagen and CFA, were measured. The data were expressed as mean ± SEM, with n = 5–6 per group. Symbols indicate statistical difference (P < 0.05, one-way ANOVA followed by Tukey’s test). ^#^P < 0.05 compared to Veh group; *P < 0.05 compared to the Veh + Challenge group.

Finally, the levels of IL-6, TNF-α, IL-17 and IFNγ in the knee joint of mice were quantified. We observed that at the dose of 10 mg/kg, neovestitol reduced IL-6 levels after 12 days of treatment (Fig. 7). Nevertheless, at 3 and 10 mg/kg it did not affect the levels of TNF-α, IL-17 and IFN-γ in the joint (Fig. 7). These results suggest that the modulatory effects of neovestitol on arthritic mice occur partly by reducing the release of IL-6.

## Discussion

Studies have reported the promising potential of isoflavonoids in the modulation of the inflammatory process^19^,^20^. A study with formononetin, another isoflavonoid isolated from Brazilian red propolis, showed that administration of this compound in experimental inflammation models reduced carrageenan-induced leukocyte migration and edema formation^21^. In another study, it was found that genistein (largely found in soy) was able to prevent inflammation in rats. The suppression of the production of pro-inflammatory cytokines such as TNF-α and IL-1β was one of the observed effects^22^. A study investigated the anti-inflammatory effects of biochanin A on BV2 microglial cells stimulated with LPS. The authors observed a reduced production of TNF-α, IL-1β, nitric oxide and reactive oxygen species^23^.

In our study, sc administration of neovestitol – an isoflavonoid isolated from Brazilian red propolis – reduced LPS-induced neutrophil migration. Overall, inflammation consists of a series of events characterized by influx of neutrophils into the inflammatory focus, and cytokines play a crucial role in this scenario^24^. Pro-inflammatory cytokines and chemokines secreted by resident macrophages perform numerous functions, including activation of endothelial cells to express adhesion proteins and activation of leukocyte locomotion mechanisms. Among these proteins, selectins (L-, P- and E-selectin) promote the interaction of white blood cells flowing in the bloodstream with endothelial cells by a rolling process. Immunoglobulins, including the intercellular adhesion molecule type 1 (ICAM-1), are responsible for the strong adhesion of leukocytes onto endothelial cells, which allows for transmigration of cells into the inflamed site^14^,^17^. In our study, the inhibitory activity of neovestitol on LPS-induced inflammation was found to be related to reduced expression of ICAM-1, and its effects did not depend upon modulation of inflammatory cytokines and chemokines. Studies have shown that nitric oxide plays a crucial role in modulating leukocyte adhesion during the inflammatory process. The action of nitric oxide (iNOS pathway) in LPS-induced inflammation is associated with a reduction in ICAM-1 expression in the mesenteric microcirculation of mice^15^,^16^. In this study, we found that treatment with an iNOS inhibitor abolished the inhibitory effect of neovestitol on ICAM-1 expression and neutrophil migration. Furthermore, it was found that treatment with neovestitol increased nitric oxide levels in the peritoneal cavity at the time of 4 hours. On the other side, the animals which received only LPS challenge showed no increase on their levels of nitric oxide. Studies show that animals subjected to inflammatory challenges (e.g. carrageenan, LPS) exhibit significantly higher levels of nitric oxide 6 hours after stimulation, in order to compensate for the exacerbation of the inflammatory process^25^,^26^. The increase in nitric oxide levels exerted by neovestitol corroborates the data on leukocyte adhesion (intravital microscopy) and ICAM-1 expression, which show an inhibitory activity of neovestitol in both events, 4 hours upon LPS stimulation. In addition, the capacity of neovestitol in directly blocking ICAM-1 expression *in vitro* (bEnd.3 cells) was evaluated. Studies have shown that some drugs have the ability to block the expression of adhesion molecules, including ICAM-1^27^,^28^. In this study, we used bEnd.3 endothelial cells, which do not show expression of iNOS and production of nitric oxide, at least within 24 h^29^. According to the *in vitro* results, cell exposure to neovestitol did not inhibit the expression of ICAM-1 directly, thus demonstrating a nitric oxide-dependent activity. Thus, it is suggested that the ameliorating effects of neovestitol on acute inflammation could be associated, at least in part, to the induction of nitric oxide (pathway iNOS → nitric oxide) by the endothelium of mesenteric microcirculation and, as consequence, reduction of leukocyte adherence due to suppression of ICAM-1.

The activity of neovestitol in collagen-induced arthritis in DBA-1/J mice was also evaluated. We found that administration of neovestitol reduced the clinical scores of animals with arthritis and the levels of IL-6. IL-6 is a cytokine involved in the inflammatory response which participates in neuronal processes and metabolism regulation^30^. It is produced by different cell types, including neutrophils, macrophages, endothelial cells, fibroblasts and dendritic cells. Its action takes place in different cell types. In neutrophils, IL-6 causes an increase in elastase. In endothelial cells, it increases the expression of ICAM-1^2^. Studies have pointed out a key role of IL-6 in the pathophysiology of rheumatoid arthritis. Among the findings, a high expression of IL-6 was detected in the synovial tissue^31^. Furthermore, the action of IL-6 in the collagen-induced arthritis model was demonstrated to be related to Th17 differentiation^32^. Here, administration of neovestitol in animals with arthritis did not change Th17 differentiation in inguinal lymph nodes. In addition, it did not affect IL-17 cytokine in the joint of animals with arthritis, although it exerted an inhibitory effect on the release of IL-6. This phenomenon could be explained by the fact that Th17 differentiation occurs early in the immunization process of the mice, as a result of the high levels of IL-6 released on the first day after the first immunization^32^,^33^. Thus, as the administration of neovestitol was performed on the 21st day once a day after the second immunization, it is suggested that its inhibitory activity on IL-6 may be associated with other types of modulation. Studies have shown that the high levels of IL-6 in the synovial fluid are also associated with joint destruction in arthritis^34^. A study found that blockage of the receptor IL-6R reduces the differentiation of osteoclasts^35^. In our study, administration of neovestitol reduced joint damage in animals with arthritis, suggesting therefore its possible influence on the inhibition of IL-6. The inhibitory effects of neovestitol on IL-6 release in the joints of mice with arthritis may be related to the increased levels of nitric oxide (iNOS pathway), as observed in the acute inflammation assays. Some studies show that nitric oxide is able to reduce NF-kB activity through a negative feedback mechanism, thus leading to decreased IL-6 release^36^,^37^. In another study, treatment with a nitric oxide synthase inhibitor after induction of iNOS expression in a chondrocyte culture led to higher degradation of IkB-α, which indicates the influence of endogenous production of nitric oxide in the inhibition of NF-κB activation^38^. Therefore, it is suggested that the reduction in IL-6 release by neovestitol may be due to an increase in the nitric oxide production (iNOS pathway), in joint tissue cells.

Altogether, the findings of our study suggest a promising activity of neovestitol in the regulation of acute and chronic inflammation. Studies have shown that the ingestion of foods that contain significant amounts of isoflavonoids can bring health benefits for the prevention of numerous chronic diseases, including cardiovascular diseases, cancer and obesity^39^,^40^. Thus, the results obtained with neovestitol are significant from the nutritional point of view, because this isoflavonoid can be included in a healthy diet in the future as a nutraceutical, in order to prevent serious chronic degenerative diseases whose etiology underlies an inflammatory process. However, further studies are needed to explore the effects of this compound on human health and disease prevention.

Thus, it is concluded that neovestitol showed inhibitory activity in LPS-induced inflammation, and its effects involve the nitric oxide pathway and consequent suppression of ICAM-1. Moreover, neovestitol inhibited the development of collagen-induced arthritis per modulation of IL-6 release. The results demonstrate a potential anti-inflammatory activity of neovestitol, an isoflavonoid that could be useful in the future for therapeutic purposes and/or incorporated as a nutraceutical compound.

## Methods

### Reagents

Ethanol, hexane, chloroform and ethyl acetate were purchased from Merck (Sao Paulo, SP, Brazil). Lipopolysaccharide (LPS), dimethyl sulfoxide (DMSO), bovine serum albumin (BSA), RPMI-1640 Medium, L-glutamine and penicillin were purchased from Sigma-Aldrich (St. Louis, MO, USA). Type II bovine collagen from MD Biosciences (St. Paul, MN, USA). Apo Screen Annexin V-FITC Kit was purchased from SouthernBiotech (Birmingham, AL, USA). CXCL2/MIP-2 from R&D Systems (Minneapolis, MN, USA). Fetal bovine serum from Gibco (Grand Island, NY, USA). Neovestitol was dissolved in 1% DMSO in PBS. The negative control group received treatment with vehicle alone (1% DMSO in PBS).

### Extraction and isolation of neovestitol

Samples of Brazilian red propolis collected by of *Apis mellifera L*. (Apidae) were obtained in Maceio (9°40′S, 35°41′W), state of Alagoas, northeastern Brazil. The chemical profile of red propolis was previously described^11^. Briefly, the propolis extract was prepared in 80% ethanol (v/v in water). The extract (39 g) was subjected to liquid-liquid fractionation and the chloroform fraction (22 g) was collected, concentrated and fractionated in a dry column using a mixture of chloroform:ethyl acetate at the ratio 7:3 as mobile phase. Subfraction 4 (2.2 g) was subjected to fractionation in Sephadex LH20 using methanol as mobile phase. The subfraction 4.2. (200 mg) was obtained and purified in semipreparative reversed-phase HPLC (Shimadzu, Ltd., Kyoto, Japan) yielding neovestitol (120 mg)^13^.

### Identification of neovestitol

**Figure 8:**
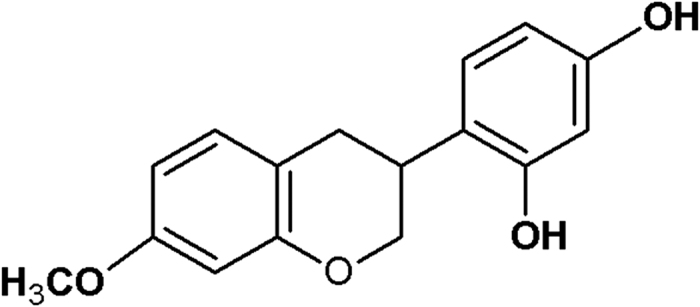
Chemical structure of neovestitol (C_16_H_16_O_4_; 272.29584 g/mol).

We used a mass spectrometry (MS) system of quadrupole-time of flight (Q-TOF) (UltrOTOF-Q, Bruker Daltonics, Bremen, Germany), with electrospray ionization (ESI) in positive and negative full scan modes. The parameters of the analysis were: capillary voltage: 3.9 KV; gas temperature: 150 °C, nebulizer gas flow: 4 L/h. The sample was added to the chromatographic system in a split mode at a ratio of 1:3. For nuclear magnetic resonance (NMR, Bruker DPX 500 MHz spectrometer, Bruker, Bremen, Germany) analysis, bioactive compounds were isolated under low pressure for complete removal of solvent and then dissolved in CD_3_OD. The ^1^H and ^13^C NMR spectra were obtained in a spectrometer at 500 and 100 MHz, respectively. Tetramethylsilane (TMS) was used as an internal standard. The data on the identification of neovestitol (2′,4′-dihydroxy-7-methoxyisoflavan) (Fig. 8) by ESI-Q-TOF-MS (see Supplementary Fig. S2) and NMR (see Supplementary Table S1) are included as supplementary information^41^,^42^.

### Endotoxin test

Endotoxin contamination in the neovestitol sample was checked using the ToxinSensor™ Single Test Kit according to manufacturer’s instructions. The kit sensitivity was >0.015 EU/mL, and the results attesting the endotoxin-free profile of neovestitol sample are included as supplementary information (see Supplementary Table S2).

### Animals

Specific pathogen-free (SPF) mice, C57BL/6 (experimental model of acute inflammation) or DBA-1/J (experimental model of chronic inflammation) weighing between 20–25 g were housed at temperatures of 22–25 °C, with a light cycle of 12 h light/12 h dark, 40–60% humidity, and with access to water and food *ad libitum*. All procedures involving the use of animals were previously approved by a local ethics committee (CEUA/Unicamp, process no. 2793-1) and methods were carried out in accordance with the approved guidelines.

### Cell culture

bEnd.3 cells, the mice brain endothelial cell line from the American Type Culture Collection (ATCC, Manassas, VA, USA), were cultured in DMEM supplemented with 10% fetal bovine serum (FBS), 2 mM of L-glutamine, 4.4 g of glucose and 100 U/mL of penicillin at 37 °C in 5% CO_2_/ 95% O_2_ atmosphere.

## Experimental model of acute inflammation

### Neutrophil migration *in vivo*

C57BL/6 mice were pretreated with neovestitol at the doses of 1, 3 or 10 mg/kg sc. The negative control group received treatment with vehicle alone (1% DMSO in PBS). After 30 min, LPS was administered intraperitoneally (ip) at 300 ng/cavity and then 6 h later the animals were killed. The peritoneal cavity of the mice was washed with 3 mL PBS/EDTA (1 mM) and total cell counting was performed in a Neubauer chamber. The differential counting was performed under optical microscopy (100x magnification) with slides prepared in a cytocentrifuge (Cytospin 4, Thermo Fisher Scientific, Waltham, MA, USA) following hematologic staining using Panotic Kit (Laborclin, Pinhais, PR, Brazil). A total of 100 cells per slide were counted and clustered in four cell types: neutrophils, eosinophils, mast cells and mononuclear cells. Quantification of each cell type was calculated as a percentage of counted cells and by the total cell number counted. Results were expressed as number of neutrophils per cavity^15^.

### Intravital microscopy

C57BL/6 mice were pretreated with neovestitol at 10 mg/kg sc. The negative control group received treatment with vehicle alone (1% DMSO in PBS). After 30 min, LPS was administered (ip) at 300 ng/cavity. The animals were anesthetized with ketamine and xylazine 2 h or 4 h after the LPS stimulus. Their mesentery was exteriorized for observation of the microcirculation (intravital microscopy DM 6000, Leica Microsystems). Leukocyte rolling (2 h after stimulus) was counted during 10 min, and a total of 3 different fields per animal were analyzed. Leukocyte adhesion (4 h after stimulus) was assessed by counting the number of leukocytes adhered to an area of 100 μm^2^ on the mesenteric venule. The results of leukocyte rolling were expressed as number of leukocytes rolling/min and the adhesion as number of adhered cells/100 μm^2^ 43,44.

### Collection of mesenteric tissue and immunofluorescence assay for ICAM-1

C57BL/6 mice were pretreated with neovestitol at the dose of 10 mg/kg sc. The negative control group received treatment with vehicle alone (1% DMSO in PBS). After 30 min, LPS was administered ip at 300 ng/cavity and 4 h later the animals were killed and their mesenteric tissue collected and frozen in Tissue-Tek (Fisher Healthcare). Sections of 5 μm of the frozen tissue were made using a cryostat (Leica Microsystems). Blocking was performed with 1% BSA. The anti-ICAM-1 antibody (BD Bioscience, San Diego, CA, USA) conjugated to FITC was incubated for 2 h. Fluoromount (SouthernBiotech, Birmingham, AL, USA) was used for attachment of the cover slip. Fluorescence images were acquired in the Leica microscope TCS SP5 (Leica Microsystems)^45^.

### Quantification of TNF-α, CXCL1/KC and CXCL2/MIP-2 *in vivo*

C57BL/6 mice were pretreated with neovestitol at the dose of 10 mg/kg sc. The negative control group received treatment with vehicle alone (1% DMSO in PBS). After 30 min, LPS was injected ip at 300 ng/cavity and, 1.5 h (quantification TNF-α) or 3 h (quantification CXCL1/KC and CXCL2/MIP-2) later the animals were killed and their peritoneal cavity washed with 3 mL PBS/EDTA. The quantification of TNF-α, CXCL1/KC and CXCL2/MIP-2 was performed by ELISA assay using protocols provided by the manufacturers (R&D Systems, Minneapolis, MN, USA). Results were expressed as pg/mL^46^.

### Administration of iNOS inhibitors on neutrophil migration

C57BL/6 mice were pretreated with aminoguanidine (Sigma-Aldrich, St. Louis, MO, USA) at 50 mg/kg sc 15 min before the administration of neovestitol at 10 mg/kg sc. The negative control group received treatment with vehicle alone (1% DMSO in PBS). After 30 min, LPS was administered ip at 300 ng/cavity and then 6 h later the animals were killed. The peritoneal cavity was washed with 3 mL PBS/EDTA (1 mM) and the total and differential cell count was performed as described earlier^15^.

### Administration of iNOS inhibitors on ICAM-1 expression

C57BL/6 mice were pretreated with aminoguanidine at 50 mg/kg sc 15 min before the administration of neovestitol at 10 mg/kg sc. The negative control group received treatment with vehicle alone (1% DMSO in PBS). After 30 min, LPS was administered ip at 300 ng/cavity and 4 h later the animals were killed and their mesenteric tissue collected and frozen in Tissue-Tek. ICAM-1 expression was evaluated by immunofluorescence as described earlier^45^.

### Quantification of nitrite

C57BL/6 mice were pretreated with neovestitol at 10 mg/kg sc. The negative control group received treatment with vehicle alone (1% DMSO in PBS). After 30 min, LPS was administered ip at 300 ng/cavity and then 4 h later the animals were killed and their peritoneal cavity washed with 3 mL PBS/EDTA. The production of nitric oxide was determined in the peritoneal lavage using the Griess method (Promega Corporation, Madison, WI, USA) at 540 nm^47^.

### bEnd.3 viability assay by MTT

bEnd.3 cells were cultured in 96-well plates (1 × 10^5^ cells/well) at 37 °C and 5% CO_2_. After complete adhesion in 24 h, the cells were pretreated with neovestitol at 30 μM and incubated for 24 h under conditions previously described. The negative control group was incubated with vehicle alone (1% DMSO in PBS). The cells were resuspended in RMPI with MTT (1 mg/mL) and incubated for 24 h. Subsequently, the supernatant was removed and 200 μL of ethanol was added. The absorbance was measured at 540 nm and 620 nm using a microplate reader (Flex Station 3 Multi-Mode Microplate Reader (Molecular Devices, Sunnyvale, CA, USA). The correction of the absorbance read at 540 nm was made by subtracting the readings at 620 nm.

### bEnd.3 cell culture

bEnd.3 cells were cultured in 6-well plates (1 × 10^6^ cells/well) at 37 °C and 5% CO_2_. Following complete adhesion during 24 h, cells were pretreated with neovestitol at 30 μM and 30 min later, LPS stimulation at 5 μg/mL was performed. The negative control group was incubated with vehicle alone (1% DMSO in PBS). Cells were kept for 24 h under conditions previously described^48^. After LPS stimulation cells were manually lysed using RIPA Buffer (Sigma-Aldrich, St. Louis, MO, USA) containing protease inhibitors and, ICAM-1 expression was determined by western blotting assay.

### Western blotting

Protein quantification was performed by the colorimetric method of Coomassie (Bradford) Protein Assay Kit (Thermo Fisher Scientific, Waltham, MA, USA). Homogenate samples were denatured at 100 °C for 10 min and applied into a polyacrylamide 10% gel using Mini-PROTEAN Tetra Cell (Bio-Rad, Hercules, CA, USA). After the run, the separating gel was transferred to a nitrocellulose membrane. The blocking of nonspecific antigenic sites was carried out by incubation of membranes for 1 hour at room temperature in Tris-buffered saline (TBS) containing 5% (w/v) BSA and 0.1% Tween 20. The ICAM-1 antibody (Santa Cruz Biotechnology, Dallas, TX, USA) and beta-actin were diluted in Tris-buffered saline (TBS) containing 5% (w/v) BSA and 0.1% Tween 20 and incubated overnight and for 1 hour, respectively. After incubation, the membranes were washed and then incubated with anti-mouse secondary antibody for 1 h at room temperature. The membranes were washed and the substrate Luminata (Millipore, Billerica, MA, USA) was added for chemiluminescence revelation using a ChemiDoc™ XRS equipment (Bio-Rad, Hercules, CA, USA) with the software Image Lab 3.0.

### Isolation of neutrophils and chemotaxis *in vitro*

Isolation of neutrophils from the bone marrow of C57BL/6 mice was performed by Percoll (Sigma-Aldrich, St. Louis, MO, USA) gradient^49^. Briefly, total cells obtained from the bone marrow (femur and tibia) of the mice, were washed and resuspended in 2 mL Hank’s buffered salt solution (HBSS, Sigma-Aldrich, St. Louis, MO, USA). Then the cell suspension was transferred to a 15 mL polypropylene sterile tubes containing 72% and 65% Percoll gradient and subjected to centrifugation (1200 at 18 °C) for 35 min. The band of neutrophil formed between the gradients was collected and the total number of cells quantified using a Neubauer chamber. The purity of neutrophils was assessed by differential count using an optical microscope (100x magnification). Approximately 95% of neutrophils were obtained after isolation.

For chemotaxis, neutrophils were incubated with neovestitol at 0.3, 3 and 30 μM at 37 °C and 5% CO_2_ for 30 min in RPMI-BSA (0.01%). The negative control group was incubated with vehicle alone (1% DMSO in PBS). The modified Boyden chamber (Neuro probe Inc., Gaithersburg, MD, USA) was used in this assay. Briefly, CXCL2/MIP-2 stimulus (30 ng/mL) was placed on one side of the membrane (5 μm pore) and 50 μL of a neutrophil suspension at 1 × 10^6^ cells/mL were placed on the other side and incubated under the same conditions described earlier. After 60 min, the membrane was removed and stained with Panotic Kit (Laborclin, Pinhais, PR, Brazil) and neutrophils were counted using an optical microscope (40x magnification). Each experiment was performed in triplicate. The results are presented as the average of neutrophils that migrated in 5 fields^50^.

### Neutrophil viability assay by flow cytometry

Neutrophils were incubated for 1.5 h under the conditions previously described with neovestitol at the concentration of 30 μM. The negative control group was incubated with vehicle alone (1% DMSO in PBS). For the viability study, the cells were resuspended in 100 μL of 1x annexin buffer. Anti-annexin V-FITC antibody (1:50) was added and incubated for 20 min at 4 °C. In addition, the anti-PI antibody (propidium iodide) (1:100) was also added to the solution at the moment of acquisition in a flow cytometer BD FACSVerse (BD Biosciences, San Diego, CA, USA). The analysis was performed using the software FlowJo^51^.

## Experimental model of chronic inflammation

### Collagen-induced arthritis

**Figure 9:**
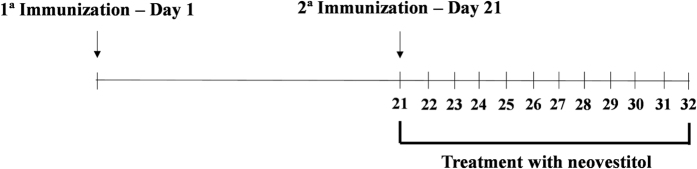
Immunization scheme with type II collagen and CFA and treatment with neovestitol.

Arthritis was induced in DBA-1/J mice by intradermal injection at the base of the tail of an emulsion containing equal volumes of type II bovine collagen and CFA (final volume 50 μL). After 21 days, the mice received a booster injection of type II bovine collagen and CFA. Treatment with neovestitol at the doses of 3 and 10 mg/kg sc, once a day, was started after the booster injection and remained for 12 days (Fig. 9). The negative control group received treatment with vehicle alone (1% DMSO in PBS). The development of experimental arthritis was monitored daily by the clinical score of the animals, as described by^52^. Score 0 (no evidence of erythema and swelling), score 1 (erythema and mild swelling confined to the tarsals or ankle joint), score 2 (erythema and mild swelling extending from the ankle to the tarsals), score 3 (erythema and moderate swelling extending from the ankle to metatarsal joints) and score 4 (erythema and severe swelling encompassing the ankle, foot and digits, or ankylosis of the limb).

### Joint histological analysis

At the end of the 12 day-treatment with neovestitol at the doses of 3 and 10 mg/kg or vehicle alone (1% DMSO in PBS), the animals were sacrificed and their whole femoral-tibial joints were collected and fixed in buffered formaldehyde 4% during 24 h before decalcification in 10% EDTA (pH = 7.2) at room temperature. The tissues were dehydrated in ethanol and embedded in paraffin following routine histological technique for hematoxylin and eosin staining. Tissues were sectioned at 7 μm and all sections were done in sagittal plane. Only slices which include entire femoral-tibial joint were chosen for subsequently analysis. Evaluation of knee joint slices was carried out using microscope coupled to digital camera (Leica Microsystems).

### Collection of draining lymph nodes and analysis of IL-17 by flow cytometry

Inguinal lymph nodes (2 per animal) were collected by a surgical procedure, then dissected, macerated in RPMI and filtered through a 100-μm sieve. The cells collected from the lymph nodes were stimulated with 50 ng/mL of phorbol-12-myristate-13-acetate and 500 ng/mL of ionomycin in the presence of BD GolgiStop (BD Bioscience, San Diego, CA, USA) for 4 h. Later, the cells were labeled and permeabilized with Perm/fix solutions (eBioscience, San Diego, CA, USA), labeled with anti-IL-17 and analyzed in BD FACSVerse (BD Biosciences, San Diego, CA, USA). Data were analyzed with FlowJo software (Tree Star, Ashland, OR, USA).

### Quantification of joint cytokines

Samples from the femoral-tibial joint were homogenized, centrifuged and the supernatant frozen at −70 °C. The quantification of IL-6, TNF-α, IL-17 and IFNγ was performed using protocols supplied by the manufacturers (R&D Systems, Minneapolis, MN, USA). The results were expressed as pg or ng/g tissue.

### Statistical analysis

The data are expressed as means ± standard error of the mean (SEM) and statistical comparison between the groups was performed by analysis of variance one-way ANOVA followed by Tukey’s test or two-way ANOVA followed by Bonferroni test. Significance was accepted when *P* < 0.05.

## Additional Information

**How to cite this article**: Franchin, M. *et al*. Neovestitol, an isoflavonoid isolated from Brazilian red propolis, reduces acute and chronic inflammation: involvement of nitric oxide and IL-6. *Sci. Rep*. **6**, 36401; doi: 10.1038/srep36401 (2016).

**Publisher’s note:** Springer Nature remains neutral with regard to jurisdictional claims in published maps and institutional affiliations.

## Supplementary Material

Supplementary Information
