# Supplementary material for: Neovestitol, an isoflavonoid isolated from Brazilian red propolis, reduces acute and chronic inflammation: involvement of nitric oxide and IL-6
Source: Sci Rep. 2016 Nov 7;6:36401. doi: 10.1038/srep36401 (PMC5098247; doi:10.1038/srep36401)
Supplement: Supplementary Information [file srep36401-s1.pdf]

**Supplementary information**

# Neovestitol, an isoflavonoid isolated from Brazilian red propolis, reduces acute and chronic inflammation: involvement of nitric oxide and IL-6

Marcelo Franchin[1], David Colon[2], Fernanda V. S. Castanheira[3], André L. L. Saraiva[3], Marcos G. Cunha[1], Bruno Bueno-Silva[1], Severino M. Alencar[4], Thiago M. Cunha[2], Pedro L. Rosalen[1,*]

[1]*Department of Physiological Sciences, Piracicaba Dental School, University of Campinas, Piracicaba, 13414 903, Brazil.*

[2]*Department of Immunology, Ribeirão Preto Medical School, University of São Paulo, Ribeirão Preto, 14049-900, Brazil.*

[3]*Department of Pharmacology, Ribeirão Preto Medical School, University of São Paulo, Ribeirão Preto, 14049-900, Brazil.*

[4]*Dental Research Division, Guarulhos University, Guarulhos, São Paulo, CEP: 07023-070, Brazil.*

[5]*Department of Agri-Food industry, Food and Nutrition, "Luiz de Queiroz" College of Agriculture, University of São Paulo, Piracicaba, 13418-900, Brazil.*

[*] Corresponding author: rosalen@fop.unicamp.br

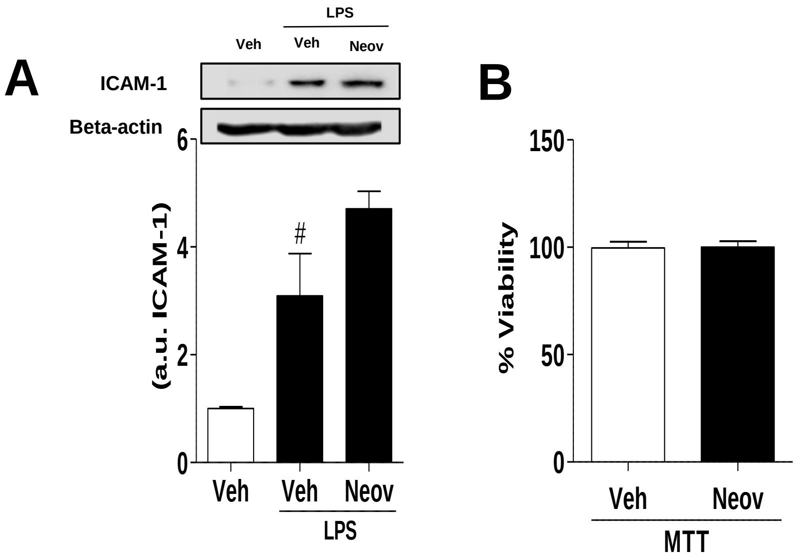


**Figure S1. Neovestitol does not affect the ICAM-1 expression in bEnd.3 cells.** bEnd.3 cells were pretreated with neovestitol (Neov) at the concentrations of 30 µM or vehicle (Veh) alone (1% DMSO in PBS), 30 min prior to LPS stimulation with 5 µg/mL. (**A**) Expression of ICAM-1 in bEnd.3 cells stimulated with LPS (5 µg/mL) for 24 h. (**B**) bEnd.3 cell viability (MTT) incubated with Neov at 30 µM for 24 h. The data were expressed as mean ± SEM, with n = 4-5 per group. Symbols indicate statistical difference ($P < 0.05$, one-way ANOVA followed by Tukey's test). # $P < 0.05$ compared to Veh group.

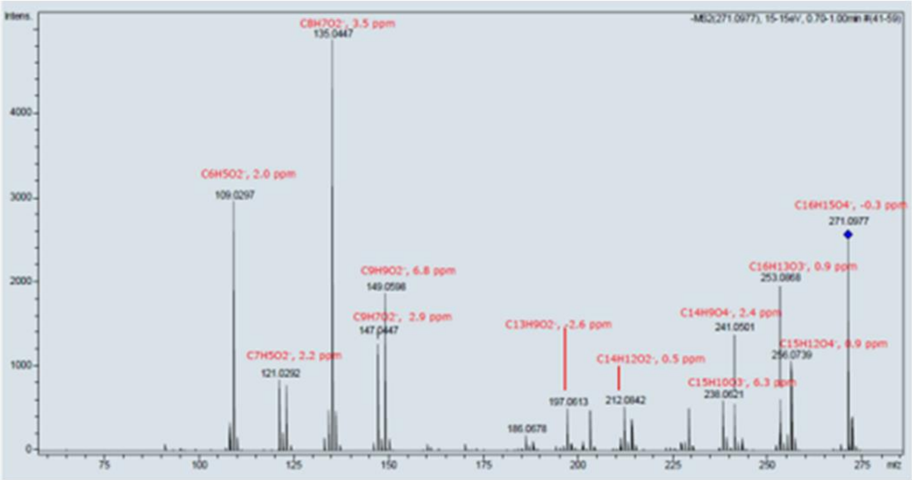


**Figure S2.** Spectrum obtained by ESI-Q-TOF-MS in negative mode of neovestitol, isolated from propolis.

**Table S1.** $^{13}$C and $^{1}$H NMR data of neovestitol (125 and 500 MHz respectively).

| Carbon atom | δ $^{13}$C | δ $^{1}$H | HMBC correlations |
|---|---|---|---|
| **2** | 69,91 | 4,04 (dd, $J_{2ax-2eq}$=10,4Hz; $J_{2ax-3}$=10,0Hz, H-2ax)<br>4,32 (ddd, $J_{2eq-4eq}$ =2,0; $J_{2eq-3}$=3,4; $J_{2ax-2eq}$=10,4, H-2eq) | H-3; H-4ax; H-4eq; |
| **3** | 31,75 | 3,50 (dddd, $J_{2eq-3}$=3,4Hz; $J_{3-4eq}$=5,5Hz; $J_{2ax-3}$=10,0Hz, $J_{3-4ax}$ =10,4Hz, H-3) | H-2ax; H-2eq; H-4ax; H-4eq; [illegible] 3´; H-6´; |
| **4** | 30,35 | 2,91 (ddd, $J_{2eq-4eq}$=2,0Hz; $J_{3-4eq}$=5,5Hz; $J_{4ax-4eq}$=15,7Hz, H-4eq)<br>2,98 (dd, $J_{3-4ax-}$=10,4Hz; $J_{4ax-4eq}$ =15,7Hz, H-4ax) | H-2ax; H-2eq; H-3; H-5 |
| **4a** | 114,41 | | H-3; H-4ax; H-4eq; H-6 |
| **5** | 130,18 | 6,98 (d, $J_{5-6}$=8,4Hz, H-5) | H-4ax; H-4eq |
| **6** | 107,29 | 6,48 (dd, $J_{5-6}$=8,4Hz; $J_{6-8}$=2,5Hz, H-6) | H-4ax; H-5; H-8 |
| **7** | 159,14 | | H-5; H-6; H-8; $OCH_3$ |
| **8** | 101,46 | 6,42 (d, $J_{6-6}$=2,5Hz, H-8) | H-4aq; H-5; H-6 |
| **8a** | 155,22(*) | | |
| **1´** | 120,08 | | H-2ax; H-2eq; H-3; H-4ax; H-4eq; H-3´; H-5´ |
| **2´** | 154,36 | | H-3; |
| **3´** | 103,13 | 6,31 (d, $J_{3´-5´}$=2,3Hz, H-3´) | H-5`; H-6´ |
| **4´** | 155,11(*) | | |
| **5´** | 108,02 | 6,38 (dd, $J$=8,3 Hz; $J_{3´-5´}$=2,3Hz, H-5´) | H-3´ |
| **6´** | 128,44 | 6,95 (d, $J_{5´-6´}$=8,3Hz, H-6´) | H-3 |
| **OCH3** | 55,35 | 3,77 | |

(*) these values may be overlapped.

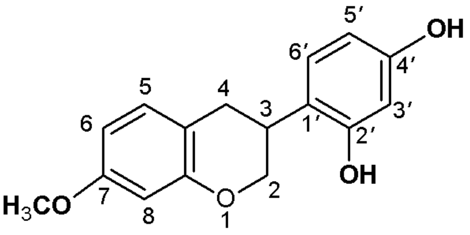

**Table S2.** Assay for verification of endotoxin contamination in the following samples: vehicle (Veh) alone (1% DMSO in PBS), LPS 300ng and neovestitol (Neov) 250 µg.

| Sample | Presence of endotoxin |
|---|---|
| Veh | - |
| LPS 300 ng | + |
| Neov 250 µg | - |

+ presence of endotoxin; - absence of endotoxin.
